# Stigma against HIV/AIDS among female sex workers and general migrant women in eastern China

**DOI:** 10.1186/s12905-014-0160-3

**Published:** 2015-01-22

**Authors:** Ying Yang, Jun Wang, Feifei Lin, Tao Zhang, Feng Yu, Yanping Zhao, Tiejun Zhang

**Affiliations:** Department of Epidemiology, School of Public Health, Fudan University, Shanghai, China and Key Laboratory of Public Health Safety (Fudan University), Ministry of Education, Shanghai, China; Minhang District Center for Diseases Control and Prevention, Shanghai, China

## Abstract

**Background:**

HIV related stigma is a recognized barrier to early detection of HIV and causes great suffering for those affected. However, data regarding HIV related stigma among female sex workers (FSW) in China was limited, with none for comparison between FSW and general migrant women (GMW). Therefore, the aim of this study was to examine HIV related stigma among FSW and GMW in Shanghai, China.

**Methods:**

A community based cross-sectional study with face-to-face interviews was conducted in Shanghai (September 2011 through December 2012), using a structured questionnaire.HIV related stigma scores were examined graphically using boxplot. A logistic regression analysis with the proportional odds model was employed to identify factors affecting HIV related stigma scores.

**Results:**

A total of 1,396 subjects, including 721 FSW and 675 GMW, were recruited in the present study. Both groups had substantial misconceptions about HIV/AIDS, although FSW had slightly higher scores on average. Both groups showed a medium level of HIV related stigma (38.34 ± 6.21 and 38.35 ± 6.86 for FSW and GMW, respectively). For the FSW, higher levels of stigma were observed for those who were in the older age groups (age 26-35 years, OR, 2.06, 95% CI 1.06-4.01), those who were married (OR, 1.62, 95% CI 1.03-2.54), and those who were working at lower-level sex service sites (OR, 1.60, 95% CI 1.06-2.43). Conversely, HIV knowledge was inversely associated with the level of HIV related stigma (OR, 0.93, 95% CI 0.87-0.98).Among GMW participating in the study, those age in the 26-35 years were more likely to show higher level of stigma (OR, 2.61, 95% CI 1.03-2.54), and HIV knowledge was found to be inversely associated with the HIV related stigma level as well (OR, 0.89, 95% CI 0.84-0.95).

**Conclusions:**

The present study suggests that there is an urgent need for the development of appropriate education strategies to reduce HIV related stigma among FSW and GMW in Shanghai, China. In particular, older women, less educated women, and women that have lived in Shanghai a relatively long time should be targeted in future stigma reduction programs.

**Electronic supplementary material:**

The online version of this article (doi:10.1186/s12905-014-0160-3) contains supplementary material, which is available to authorized users.

## Background

HIV-related stigma refers to “prejudice, discounting, discrediting directed at people perceived to have HIV/AIDS, as well as individuals, groups and communities with which they are associated” [1]. It has a profound effect on people’s decisions and behaviors, and can reduce people’s willingness to engage in HIV testing, treatment, and prevention. Since the beginning of the HIV/AIDS epidemic over 30 years ago, stigma has been a barrier to HIV testing, diagnosis, treatment and care [2-5]. Although the Chinese government has been putting an enormous effort into the HIV prevention for decades [6], stigmatization against people living with HIV/AIDS (PLWHA) is still perpetuated nationwide [5,7-10].

China has experienced a rapid increase in the incidence of HIV. Annual reports indicate a steady increase in new HIV infections over the past two to three decades [11,12]. According to the Joint United Nations Programme on HIV/AIDS (UNAIDS) 2010 report on the global AIDS epidemic, it was estimated that 740,000 people were living with HIV/AIDS in China [13]. Sexual contact has been found to be a major route of HIV transmission in China [13], and female sex workers (FSW) are at high risk of HIV infection. Based on data from 11 studies, FSW were more likely to be infected by HIV (3.0%) compared with in all other women of reproductive age (0.06%), with an odds ratio of 50.0 (95% CI 46.0–54.4) [14]. FSW bear a disproportionate burden of HIV in China, accounting for almost half (48.6%) of all HIV cases among women in that country [14]. Yet they continue to be excluded, sometimes systematically, from HIV services because of HIV/AIDS related stigma, discrimination, and criminalization.

In the past two decades, market-oriented reforms have radically changed the social landscape of China. One of the most significant developments has been the unprecedented growth in transient rural to urban migration. There have been more than 230 million migrants, approximately 17% of the total population in China. Uniquely, FSW are a marginalized sub-group of women as well as a marginalized sub-group of migrants [15]. Due to their limited education and skills, these women have few job opportunities. Unable to find a source of income in the cities, some women engage in commercial sex work to earn a living, thus becoming vulnerable to contracting HIV [15-19]. Many of the HIV-infected individuals in China are believed to be among the nation’s 230 million rural-to-urban migrants. Correspondingly, they could have played important roles in HIV transmission in China.

There are several knowledge gaps in the existing literature regarding HIV related stigma. First, few studies have examined the HIV related stigma among FSW. Second, most of the previous studies were conducted among HIV positive individuals, and the HIV related stigma among FSW has not been well documented. Although the majority of FSW are migrants, the differences regarding HIV/AIDS related stigma between FSW and general migrant women (GMW) have not been well explored. To date, no study has yet targeted two kinds of subpopulations together. Interestingly, the two types of subjects share some characteristics, such as frequent moving, unstable work or away from family. Furthermore, these two types of workers are loosely organized and offering HIV education programs to them is understandably difficult. Therefore, in the present study, we compared HIV related stigma and HIV knowledge between FSW and GMW, and examined factors affecting HIV related stigma.

## Methods

### Study setting and participants

The present study was conducted during the period from September 2011 to December 2012 in Minhang District, Shanghai, one of the most developed metropolises in China. The first HIV case in Shanghai was reported in 1987. By the end of 2012, 7,647 HIV/AIDS cases had been reported, 90% of the newly identified cases were sexually transmitted (Shanghai CDC HIV/STDs surveillance data, 2012).

For purposes of this study, a migrant woman was defined as someone who was born and registered as a permanent resident in a rural area outside of Shanghai but was living in Shanghai at the time when the survey was conducted. A female sex worker was a woman who has regularly exchanged sex services for money in commercial establishments such as karaoke bars or nightclubs for more than 6 months.

Thus far, two groups of women were recruited into the current study. Female sex workers were those who: 1) were aged between 18 and 65 years; 2) had been conducting commercial sex activities; 3) had been living in the community for more than 3 months; 4) had no official status of local residency; and 5) signed an informed consent. General migrant women were those who: 1) were aged between 18 and 65 years; 2) had been living in the community for more than 3 months; 3) had no official status of local residency; and 4) signed an informed consent. All the participants were confirmed to be HIV negative by laboratory tests.

### Procedures

A multiple stage cluster sampling method was applied to recruit the participants. In the first stage, three out fourteen communities were randomly selected. Because commercial sex is still illegal in China, the majority of FSWs work in entertainment venues or personal service sectors such as karaoke bars, nightclubs, and massage parlors. To obtain FSW participants for the study, venues such as karaoke bars and massage parlors that were known by the local public health agency to provide commercial sex services were listed and selected for sampling. Female sex workers at the selected venues were approached and asked to participate in the study. The procedures for selecting GMW as participants were different. A variety of venues (e.g. factories, markets) that employed GMW were selected. GMW working in these places were enrolled in the study. In general, sample selection gave priority to small-to-medium-sized establishments to prevent over representation by large ones in the given sample size. Research staff then made a visit to all the selected venues and approached all potential subjects individually, explaining the purpose of the study and how the information collected would be used. Initially, A total of 1,500 subjects, including 750 FSW and 750 GMW, were approached, and 1,396 (93.1%) agreed to participate in the survey.

Anonymous interviews were administered in private rooms. Based on a pilot qualitative study, the face-to-face interviews were preferred by participants. All the eligible individuals included in the study were interviewed by trained female physicians or female post-graduate students who had previous experience of administering epidemiological questionnaires. Interviewers were trained in the conceptual framework of the study, technical aspects of its implementation, and the ethical parameters involved in social research.

Ethical approval for the study was issued by the Ethics Committee of the School of Public Health, Fudan University. The nature and purpose of the study were explained to each participant, after which they were asked to sign their consent to participate in the survey. A copy of the signed consent form was given to the participant. All participants received cash incentives (US$8) for participating and travel expense reimbursements.

### Measures

The instrument design was guided by a literature review, key informants, and input from experts. The questionnaire was pilot-tested and refined prior to use, and was administered to collect information on demographic characteristics, knowledge of HIV/AIDS, HIV related stigma and perceptions of the availability of HIV/AIDS and STD related information and services etc.

Participants’ prejudicial attitudes toward PLWHA were assessed by asking participants their level of agreement with statements such as “A person with HIV must have done something wrong and deserves to be punished”, or “A person who has HIV is dirty”. A total of 12 statements were used, and responses were coded on a 1 (strongly disagree) to 5 (strongly agree) scale (Cronbach’s alpha = 0.80).

Stigma is a complex issue that has its roots in many domains, including culture, economics, knowledge, and policy. Based on some highly relevant literatures [20-24], HIV related stigma was thought to be derived from socio-cultural values, morals, and intrapersonal emotions such as negative feelings, beliefs, and attitudes regarding PLWHA. These items, as presented in the structured questionnaire used in the study, were classified into four conceptual domains (see Additional file 1: Table A1). We believe these four domains are practically useful for evaluating the HIV related stigma among the study participants, at both the individual level and at the community level. The four conceptual domains are as follows:

1. Community or personal norms towards PLWHA
2. Blame for PLWHA
3. Sympathy and support to PLWHA
4. Fear of transmission and disease

The HIV-related stigma score was constructed by taking the sum of all 12 items, therefore, summary scores could range from 12 to 60 (highest degree of stigma). The direction of some items was reversed so that a higher score consistently indicated a higher degree of general prejudicial attitude. The stigma score was analyzed as an ordinal response with three levels: low (score ≤ 30), medium (30 < score ≤ 45), and high (score >45).

Knowledge of HIV/AIDS was assessed with 12 questions that have been used previously, together or separately, in many HIV studies to measure HIV-related knowledge [25-27]. With regard these questions, six questions were about the three transmission modes of HIV and condom use for HIV prevention, three regarding misconceptions about HIV transmission through mosquito bites, shaking hands and eating with an HIV-infected individual, two about HIV diagnosis and antibiotic usage, and one on whether HIV/AIDS was curable. For each item, a correct answer was given 1 point; stating “don’t know” and incorrect answer received 0 points. Total scores could therefore range from 0 to 12 (Cronbach’s alpha = 0.78).

### Statistical analysis

Data from the questionnaires were recorded in duplicate using EpiData 3.0 for Windows (The EpiData Association, Odense, Denmark). Data validation tools were also used to validate the duplicate data entry. The database was then transferred into Statistical Package for Social Science (SPSS) software 20.0 (SPSS Inc., Chicago, IL) for statistical analysis. Differences for demographic variables were tested using chi-square and t-test for categorical and continuous variables, respectively. To describe the response pattern of each individual HIV-related stigma item, the frequencies of each question were tabulated by groups. The difference in the HIV-related stigma scores between FSW and GMW groups was examined graphically by using boxplot. A boxplot is a convenient way of graphically depicting groups of numerical data through summaries of stigma scores. Multiple ordinal logistic regression was conducted to determine factors associated with increasing level of HIV related stigma. Odds ratios (ORs) and 95% confidence intervals (CIs) were calculated. All statistical tests were two sided, and a *p* value less than 0.05 was considered statistically significant.

## Results

### Participant characteristics

A total of 1,396 subjects, including 721 female sex workers and 675 general migrant women, were finally recruited for the present study. Demographic characteristics of the participants are shown in Table 1. Among the FSW, the mean age of all participants was 26.5 ± 7.08 years. They were predominantly Han ethnicity (94.5%), 56.0% of them were aged18-25 years, 57.3% were single or widowed, 23.7% had a high school education or above, and 19.8% of them reported having monthly income more than 4000 Yuan. Approximately, 51.2% of them had lived in Shanghai for less than one year and 46.7% had migrated to more than 2 cities. Among the GMW, participants had a mean age of 29.4 ± 8.4 years, 43.7% of them were aged18-25 years, a majority (96.0%) of them were Han ethnicity, 64.1% were married, 34.7% had a high school education or above, and very few (1.5%) reported monthly income more than 4000 Yuan. More than half of them had lived in Shanghai for less than one year and nearly half of them had migrated to more than 2 cities.

**Table 1:** **Socio-demographic characteristics of study participants: 721 female sex workers (FSW) and 675 general migrant women (GMW), Minhang District, Shanghai, China, 2011–2012**

**Characteristics**	**FSW (n = 721)**	**GMW (n = 675)**	**Total (n = 1396)**
	**N (%)**	**N (%)**	**N (%)**
**Age group (p < 0.001)**			
18-25 years	404 (56.0)	295 (43.7)	699 (50.1)
26-35 years	229 (31.8)	194 (28.7)	423 (30.3)
≥36 years	88 (12.2)	186 (27.6)	274 (19.6)
**Ethnicity (p = 0.176)**			
Han	681 (94.5)	648 (96.0)	1329 (95.2)
Other	40 (5.5)	27 (4.0)	67 (4.8)
**Duration in Shanghai (p = 0.019)**			
<1 year	369 (51.2)	303 (44.9)	672 (48.1)
≥1 year	352 (48.8)	372 (55.1)	724 (51.9)
**Marital status (p < 0.001)**			
Single/widowed	429 (57.3)	242 (35.9)	671 (48.1)
Married	292 (42.7)	433 (64.1)	725 (51.9)
**Education (p < 0.001)**			
Elementary school or lower	128 (17.8)	85 (12.6)	213 (15.3)
Middle school	422 (58.5)	356 (52.7)	778 (55.7)
High school or higher	171 (23.7)	234 (34.7)	405 (29.0)
**Monthly income (Yuan, p < 0.001)**			
≤2000	120 (16.6)	389 (57.6)	509 (36.5)
2001-4000	458 (63.6)	276 (40.9)	734 (52.6)
≥4001	143 (19.8)	10 (1.5)	153 (10.9)

*Abbreviations:* *FSW* female sex workers, *GMW* general migrant women.

Chi-square test was applied for the comparison of these characteristics; P values less than 0.05 were considered statistically significant.

Compared with the GMW group, FSW were significantly younger (26.5 ± 7.08 years vs. 29.4 ± 8.4 years, P < 0.001), and were more likely to have a shorter duration in Shanghai, and to be unmarried and less educated. Likewise, FSW were more likely to earn a higher income. There was no significant difference in ethnicity between the two groups.

### HIV/AIDS related knowledge

Scores on HIV/AIDS related knowledge ranged from 0 to 12. Both FSW and GMW had a substantial misconception regarding HIV transmission, prevention, diagnosis and treatment, and FSW had a slightly but significantly higher score than GMW (8.21 ± 2.8 vs. 7.64 ± 2.8, p < 0.001). The most serious misconceptions concerned the transmission possibility of mosquito bites and the use of condoms for HIV prevention. Approximately 34.3% of FSW and 37.5% GMW incorrectly reported that HIV could be transmitted via mosquito bites. At the same time, only 70.5% of FSW and 54.4% GMW correctly reported that condom use can prevent HIV transmission.

### HIV related stigma

**Figure 1:**
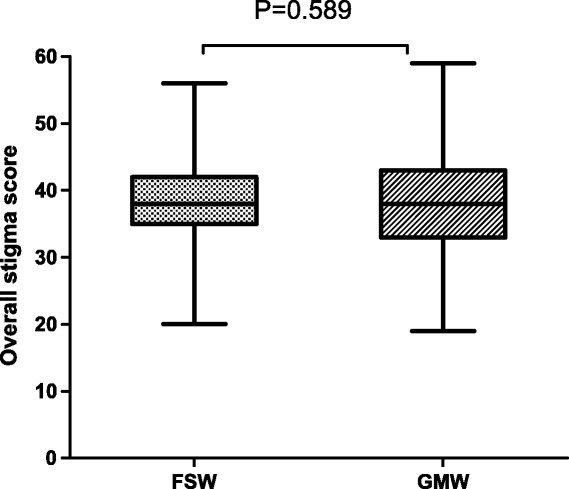
**Boxplots of HIV stigma score for female sex workers (FSW) and general migrant women (GMW).**

Both groups of women displayed a medium level of HIV related stigma. The mean of general prejudicial attitudes was 38.34 ± 6.21 and 38.35 ± 6.86 for FSW and GMW, respectively. The summed scores ranged from 20 to 50 among FSW and from 19 to 50 among GMW. No difference was detected between the FSW and GMW groups in the stigma scores (Figure 1). The stigma was further categorized into four domains: Domain (1) mean score 9.38 ± 2.08 *vs*. 9.33 ± 1.99 for FSW and GMW, p = 0.652, Domain (2) mean score 12.39 ± 2.8 *vs*. 12.94 ± 2.91 for FSW and GMW, p < 0.001, (3) mean score 5.98 ± 1.34 *vs*. 5.77 ± 1.47 for FSW and GMW, p = 0.05 (4) mean score 10.58 ± 2.31 *vs*. 10.57 ± 2.28 for FSW and GMW, p = 0.903.

Table 2 shows the results from descriptive analyses of HIV related stigma measures by four domains. A significant difference between FSW and GMW was observed for the statements such as “People who get HIV/AIDS through sex or drug use get what they deserve” and “AIDS is a punishment for bad behavior”. Similar patterns were observed with the statements that measured the stigmatizing attitudes. Conversely, no significant difference was detected between the two groups in other statements such as, “People living with HIV should have the right to marry” and “You are afraid of people living with HIV”. Overall, HIV related stigma was prevalent in both groups, and a large proportion of the study participants were afraid of people living with HIV.

**Table 2:** **Comparisons of HIV related stigma measures between 721 female sex workers and 675 general migrant women in Shanghai from 2011-2012**

**Domain**	**FSW (n = 721)**	**GMW (n = 675)**
	**N (%)**	**N (%)**
***Domain One: Community or personal norms towards people living with HIV***		
**You would feel ashamed if someone you know got HIV/AIDS (p = 0.008)**		
Agree	256 (35.5)	203 (30.1)
Not sure	227 (31.5)	196 (29.0)
Disagree	238 (33.0)	276 (40.9)
**You would feel ashamed if someone in your family got HIV/AIDS (p = 0.161)**		
Agree	285 (39.5)	240 (35.6)
Not sure	188 (26.1)	171 (25.3)
Disagree	248 (34.4)	264 (39.1)
**You would be willing to work with an HIV-positive coworker (p = 0.499)**		
Agree	173 (24.0)	174 (25.8)
Not sure	217 (30.1)	212 (31.4)
Disagree	331 (45.9)	289 (42.8)
***Domain Two: Blame for people living with HIV***		
**AIDS is a punishment for bad behavior (p = 0.015)**		
Agree	242 (33.6)	263 (38.9)
Not sure	267 (37.0)	202 (30.0)
Disagree	212 (29.4)	210 (31.1)
**People who behave promiscuously should be blamed for AIDS (p < 0.001)**		
Agree	301 (41.7)	457 (67.7)
Not sure	263 (36.5)	124 (18.4)
Disagree	157 (21.8)	94 (13.9)
**People who get HIV/AIDS through sex or drug use, get what they deserve (p < 0.001)**		
Agree	262 (36.3)	312 (46.2)
Not sure	263 (36.5)	199 (29.5)
Disagree	196 (27.2)	164 (24.3)
**People who have HIV are dirty (p < 0.001)**		
Agree	202 (28.1)	189 (28.0)
Not sure	306 (42.4)	208 (30.8)
Disagree	213 (29.5)	278 (41.2)
***Domain Three: Sympathy to people living with HIV***		
**People living with HIV should have the right to marry (p = 0.072)**		
Agree	345 (42.0)	271 (40.1)
Not sure	274 (33.4)	203 (30.1)
Disagree	202 (24.6)	201 (29.8)
**People with HIV should lose their jobs (p = 0.006)**		
Agree	226 (31.3)	178 (26.4)
Not sure	287 (39.8)	249 (36.9)
Disagree	208 (28.9)	248 (36.7)
***Domain Four: Fear of transmission and disease***		
**You are afraid of people living with HIV/AIDS (p = 0.098)**		
Agree	495 (68.7)	432 (64.0)
Not sure	148 (20.5)	147 (21.8)
Disagree	78 (10.8)	96 (14.2)
**You would not buy anything from a food vendor who has HIV/AIDS (p = 0.127)**		
Agree	379 (52.6)	387 (57.3)
Not sure	184 (25.5)	166 (24.6)
Disagree	158 (21.9)	122 (18.1)
**You would not share eating utensils with a person living with HIV because you are afraid of getting infected (p = 0.836)**		
Agree	418 (57.9)	382 (56.6)
Not sure	166 (23.1)	157 (23.2)
Disagree	137 (19.0)	136 (20.1)

*Abbreviations:* *FSW* female sex workers, *GMW* general migrant women.

The Chi-square test was applied for the comparison of these characteristics, p values less than 0.05 were considered statistically significant.

### Correlates of HIV related stigma among study participants

An ordinal logistic regression was used to determine the factors associated with stigma scores (low (score ≤ 30), medium (30 < score ≤ 45), and high (score >45)) (Table 3). With regard to the FSW group, results indicated that those who were in the older age group were more likely to report prejudicial attitude (age 26-35 years, OR, 2.06, 95% CI 1.06-4.01). Those living in Shanghai for a year or more were more likely to report negative attitudes than those who had lived in Shanghai less than 1 year (OR, 1.37, 95% CI 0.96-1.97), although the association was marginal. In addition, married FSW were more likely to manifest a higher level of stigma than those who were single (OR, 1.62, 95% CI 1.03-2.54). Moreover, those who working in some lower level sex service sites were more likely to show stigma attitude (OR, 1.60, 95% CI 1.06-2.43). Conversely, HIV knowledge was inversely associated with HIV related stigma level (OR, 0.93, 95% CI 0.87-0.98).

**Table 3:** **Potential risk factors for HIV related stigma among female sex workers (n = 721) and general migrant women (n = 675)**

**Variables**	**FSW (n = 721)**			**GMW (n = 675)**		
	**OR**	**95% CI**	**P-value**	**OR**	**95% CI**	**P-value**
**HIV knowledge**	0.93	0.87-0.98	**0.019**	0.89	0.84-0.95	**<0.001**
**Age (years)**						
18-25	Ref	--	--	Ref	--	--
26-35	2.06	1.06-4.01	**0.033**	2.61	1.49-4.55	**0.001**
≥36	1.06	0.57-1.98	0.853	1.53	0.96-2.45	0.074
**Education level**						
Elementary or low	Ref	--	--	Ref	--	--
Middle school	0.15	0.46-1.51	0.531	0.85	0.45-1.60	0.604
High school or higher	1.08	0.69-1.68	0.731	0.83	0.56-1.24	0.361
**Ethnicity**						
Han	Ref	--	--	Ref	--	--
Minority	1.38	0.66-2.99	0.375	1.46	0.63-3.39	0.379
**Marital status**						
Single/widowed	Ref	--	--	Ref	--	--
Married	1.62	1.03-2.54	**0.036**	1.64	0.82-3.12	0.160
**Living with spouse or a sexual partner**						
Yes	Ref	--	--	Ref	--	--
No	0.92	0.62-1.36	0.686	1.25	0.70-2.25	0.453
**Perceived HIV risk**						
Yes	Ref	--	--	Ref	--	--
No	0.75	0.38-1.48	0.403	0.73	0.34-4.56	0.413
**Heard of VCT**						
Yes	Ref	--	--	Ref	--	--
No	0.16	0.54-1.28	0.407	1.25	0.85-1.97	0.224
**Monthly income (Yuan)**						
≤2000	Ref	--	--	Ref	--	--
2001-4000	0.91	0.47-1.93	0.777	0.74	0.12-3.01	0.672
≥4001	1.48	0.91-2.43	0.114	0.57	0.14-2.31	0.431
**Duration in Shanghai**						
<1 year	Ref	--	--	Ref	--	--
≥1 year	1.37	0.96-1.97	0.086	1.23	0.87-1.73	0.235
**Having tested HIV**						
Yes	Ref	--	--	Ref	--	--
No	1.08	0.63-1.84	0.780	0.98	0.17-5.77	0.998
**Sex working sites**						
High level	Ref	--	--	N/A	N/A	N/A
Low level	1.60	1.06-2.43	**0.026**	N/A	N/A	N/A

*Abbreviations:* *FSW* female sex workers, *GMW* general migrant women.

Ordinal logistic regression analysis was performed. OR = odds ratio, CI = confidence interval, p-values less than 0.05 were considered statistically significant.

Results for GMW indicated that those in the older age group (age 26–35 years) were more likely to express a higher level of stigma (OR, 2.61, 95% CI 1.03-2.54), and HIV knowledge was found to be inversely associated with the level of HIV related stigma as well (OR, 0.89, 95% CI 0.84-0.95).

## Discussion

HIV/AIDS related stigma has been a barrier to HIV/AIDS testing, treatment and care, since the beginning of the HIV/AIDS epidemic in China 25 years ago [4,5,26,28,29]. The differences in HIV related stigma between FSW and GMW have not been previously investigated in China. Information from the present study may benefit programs targeting reduction of HIV/AIDS stigma among FSW and GMW in this country.

The current study identified a medium level of HIV/AIDS stigma in both groups of participants. These findings are consistent with previous studies [30-33], and indicate that negative feelings may be widespread among various populations towards someone with HIV, suggesting that this is an important public health issue that needs to be urgently addressed. Our finding that approximately 70% of the participants were afraid of HIV infected individuals strongly suggests an urgent need to initiate health education programs to reduce discrimination towards PLWHA. Interestingly, FSW showed a somewhat lower level of HIV/AIDS related stigma in specific stigma items compared with GMW, although no significant differences were detected with regard to the summary scores of HIV related stigma. A better acceptability among FSW was noted in responses to some of the questions. For example, regarding the statement, “People who get HIV/AIDS through sex or drug use get what they deserve”, only 36.3% of FSW said “yes”, compared with 46.2% of GMW; likewise, for the statement, “People who behave promiscuously should be blamed for AIDS”, the proportion of FSW agreeing was 41.7%, compared with 67.7% for GMW. These results are in accordance with those from a previous study [8,34].

The present study showed that HIV/AIDS knowledge was inversely associated with HIV/AIDS related prejudicial attitudes, which are consistent with the results from previous studies [8,25,35]. Thus far, the HIV/AIDS knowledge may cast an impact on the level of HIV/AIDS related stigma [36]. Of note, despite increasing concern about HIV/AIDS among public health officials in China, widespread misconceptions exist among both groups about HIV transmission [37,38]. Similarly, substantial misconceptions regarding HIV/AIDS knowledge were detected among study participants in the present study. The co-existence of correct and incorrect knowledge has been documented in other settings and may reflect preexisting misunderstandings of disease transmission combined with more recent exposure to HIV prevention information [39,40]. Moreover, a slightly higher HIV/AIDS knowledge level was detected among FSW compared with GMW. Based on these findings, stigma reduction intervention programs should consider giving a priority to the delivery of HIV/AIDS knowledge to both FSW and GMW, which might help reduce HIV related stigma.

The present study also showed that older women were more likely to report a negative attitude. This result is consistent with previous studies that less stigma was reported among person under 30 years of age [8,22,41,42]. It suggests that older women were more likely to hold negative attitude towards PLWHA. In the current study, women who had been living in Shanghai longer and women who were married were more likely to show a stigma attitude. These women may have a more stable living situation, compared with those who have lived in Shanghai for shorter periods, and therefore may have higher economic status. Data have shown that more than half of the participants, in either the FSW or GMW group, migrated frequently across different cities. Their migrant status is also of importance in the HIV/AIDS prevention program, which deserves extensive research. Therefore, HIV/AIDS stigma reduction programs should be specifically designed by taking these factors into consideration. Moreover, those FSW from lower level sex service site were more likely to report higher HIV-related stigma. It indicates that stigma reduction interventions among these specific groups are highly warranted.

This study has allowed us to address previously unexplored questions in the context of a relatively large, representative population. However, the study has several limitations. First, due to the observational nature of the data, it could limit causal inference ability. Second, self-reported data are subject to self-reporting bias. Female sex workers whose activities are still regarded as illegal in China may provide a socially desirable answer, which may underestimate the stigma attitude. Third, we analyzed the potential correlates for HIV related stigma based on prior knowledge and the available literature, which may not comprehensive. Finally, respondents were recruited from a relatively prosperous metropolitan area in China. This aspect of the sample may restrict its generalizability; however, it is likely that the characteristics of the study participants reflect the situation in similar settings in China.

The present study provides new evidence that HIV/AIDS related stigma is prevalent among both FSW and GMW in Shanghai, China. HIV/AIDS-related public health interventions are needed to facilitate reduction and eventual elimination of HIV/AIDS-related stigma in both populations.

## Conclusions

The present study suggests an urgent need for the development of appropriate education strategies to reduce HIV related stigma for women, particularly those who are older, less educated, and have lived in Shanghai relatively longer. Both FSW and GMW should be targeted in future stigma reduction programs.

## Additional file

Additional file 1: Table A1. Selected Conceptual Domains and Questions/Probes in the Structured Interview Guide.
