# Supplementary material for: Stigma against HIV/AIDS among female sex workers and general migrant women in eastern China
Source: BMC Womens Health. 2015 Jan 22;15:2. doi: 10.1186/s12905-014-0160-3 (PMC4316807; doi:10.1186/s12905-014-0160-3)
Supplement: Additional file 1: Table A1. — Selected Conceptual Domains and Questions/Probes in the Structured Interview Guide. [file 12905_2014_160_MOESM1_ESM.docx]

**Additional file 1**

**Stigma against HIV/AIDS among female sex workers and general migrant women in eastern China**

Ying Yang, Jun Wang, Feifei Lin, Tao Zhang, Feng Yu, Yanping Zhao, Tiejun Zhang

**Contents:**

Table A1. Selected Conceptual Domains and Questions/Probes in the Structured Interview Guide

**Table A1. Selected Conceptual Domains and Questions/Probes in the Structured Interview Guide**

| Domain | Questions/Probes in Interview Guide |
| --- | --- |
| Community or personal norms towards people living with HIV/AIDS (PLWHA) | 1. You would feel ashamed if someone you know got HIV/AIDS  2. You would feel ashamed if someone in your family got HIV/AIDS  3. You would be willing to work with an HIV-positive coworker |
| Blame for PLWHA | 1. AIDS is a punishment for bad behavior  2. People who behave promiscuously should be blamed for AIDS  3. People who get HIV/AIDS through sex or drug use, get what they deserve  4. Person who has HIV is dirty |
| Sympathy and support to PLWHA | 1. People living with HIV should have the right to marry  2. People with HIV should lose their jobs |
| Fear of transmission and disease | 1. You would not share eating utensils with a person living with HIV because you are afraid of getting infected  2. You are afraid of people living with HIV/AIDS  3. You would not buy anything from a food vendor who has HIV/AIDS |
